# Association of menstrual phase with smoking behavior, mood and menstrual phase-associated symptoms among young Japanese women smokers

**DOI:** 10.1186/1472-6874-13-10

**Published:** 2013-03-02

**Authors:** Hiroko Sakai, Kazutomo Ohashi

**Affiliations:** 1 Department of Nursing, Faculty of Health Sciences, Morinomiya University of Medical Sciences, Osaka, Japan; 2 Department of Children and Women’s Health, Division of Health Sciences, Osaka University Graduate School of Medicine, Osaka, Japan

**Keywords:** Menstrual phase, Smoking, Craving, Depressiveness, Young Japanese women

## Abstract

**Background:**

Previous studies of the relationship between the menstrual phases and smoking behavior have been problematic, so the association of menstrual phases with smoking behavior and correlations among smoking, psychological and physical conditions in each phase of the menstrual cycle are unclear.

**Methods:**

To accurately examine the association between menstrual phases and the amount of smoking (number of cigarettes smoked and breath CO concentration), craving of smoking on visual analogue scale (VAS), depression in the Center for Epidemiologic Studies Depression (CES-D) Scale, and menstrual phase-associated symptoms in the Menstrual Distress Questionnaire (MDQ), we improved various methodological issues, specifically, 1) Ovulation was confirmed by measuring the basal body temperature and identifying a urinary luteinizing hormone (LH) surge in two cycles; 2) The menstrual, follicular, and luteal phases were clearly defined for subjects with different menstrual cycles; 3) The breath CO concentration was measured every day. A notice was posted on public bulletin boards to recruit research subjects and twenty-nine young Japanese women smokers aged 19 to 25 years old were analyzed.

**Results:**

The number of cigarettes smoked was greater and the CO concentration was higher in the luteal phase than in the follicular phase. The levels of craving for smoking (VAS), depressiveness (CES-D), and menstrual phase-associated symptoms (MDQ) in the menstrual and luteal phases were higher than those in the follicular phase. The mean score for CES-D was 16 points (the cut-off value in screening for depression) or higher in the menstrual (16.9 ± 8.2) and luteal phases (17.2 ± 8.4).

The number of cigarettes smoked and CO concentration were significantly correlated with the levels of craving for smoking, depressiveness, and menstrual phase-associated symptoms in all phases except for MDQ scores in follicular phase. The amount of smoking in the luteal phase was most strongly correlated with these symptoms.

**Conclusions:**

In the menstrual and luteal phases, young Japanese women smokers increased their amount of smoking and suffered from greater craving for smoking, depressiveness and menstrual phase-associated symptoms. The amount of smoking was correlated with these symptoms, but their cause-effect relationship has not been determined yet.

## Background

Females of reproductive age develop a variety of physical and psychological symptoms in the menstrual and luteal phases, which may require medical intervention in serious cases [1]. It has been considered that women smoking behavior fluctuates across the menstrual cycle and that women may smoke to alleviate symptoms associated with the menstrual cycle. Since the 1980s, a large number of studies have been conducted on the relationship between the menstrual cycle and changes in the amount of smoking. The studies [2-4], excluding one [5], published in the first half of the 1990s or earlier reported an increase in the amount of smoking in the luteal phase. However, in most of these studies, the basal body temperature or hormone level in the blood was not measured to accurately define each phase of the menstrual cycle. Several studies conducted in the 1990s tried to define the phases by measuring blood hormone levels and detecting a urinary luteinizing hormone (LH) surge to confirm ovulation [6-10]. Furthermore, although these studies measured concentrations of breath carbon monoxide (CO) and urinary cotinine, a metabolite of nicotine, to objectively assess the amount of smoking, they did not provide consistent results regarding the relationship between the amount of smoking and the menstrual cycle. According to a study conducted by DeBon et al. [8], there was an increase in the number of cigarettes smoked in the menstrual and luteal phases compared to the ovulatory phase, although no changes were noted in breath CO and urinary cotinine levels. In contrast, Allen et al. [6,7], reported no marked changes in the number of cigarettes smoked and breath CO and urinary cotinine levels between each phase of the menstrual cycle. Our previous study [11] reported that young Japanese women smokers had more severe menstrual phase-associated (menstrual and premenstrual) symptoms than non-smokers and that the severity of menstrual phase-associated symptoms was markedly correlated with nicotine dependency and motivation for smoking.

It has been pointed out that previous studies of the relationship between the menstrual cycle and smoking involved a variety of problems. In their review of the relationship between smoking and the menstrual cycle, Carpenter et al. [12] suggested that in most of the previous studies confirmation of ovulation was insufficient, the follicular and luteal periods were not clearly defined, and objective indices (blood and urinary cotinine concentrations and breath CO concentration) were not used to assess the daily amount of smoking. Secondly, patients with depression, dysmenorrhea, and premenstrual syndrome were not excluded from research subjects, which presumably affected the research results. Furthermore, as the subjects of previous studies were women in their 20s to 40s, there might have been age-related differences in symptoms associated with the menstrual cycle and smoking status.

By addressing the problems described above, the present study involving young Japanese women examined the changes of the amount of smoking among menstrual phases and the relationships between the amount of smoking versus the levels of craving for smoking, depressiveness, and menstrual phase-associated symptoms in the menstrual, follicular, and luteal phases.

## Methods

### Participants

The study was conducted between August 1, 2009 and April 15, 2010. A notice was posted on seven bulletin boards in the Osaka City Health Center and Aino University to recruit research subjects from among women smokers in their twenties. The researcher provided each applicant with verbal and written explanations of the purpose of the study and the method for the completion of a study form in a private room, and obtained written consent.

The subjects were 33 women who provided written consent. An electronic thermometer and instrument for measuring breath CO concentrations were lent to the subjects. Question items included age, occupation, the number of cigarettes smoked each day, and days of menstrual cycle and days of menses during the past three months. The level of nicotine dependence was determined by the Fagerström Test for Nicotine Dependency (FTND) [13]. The FTND consists of 6 items and its score is calculated as the total sum of scores. The total score ranges from 0 to 10 and a higher score indicates higher nicotine dependency. Scores 0 to 3 indicate mild dependency, scores 4 to 6 indicate moderate dependency, and scores 7 to 10 indicate severe dependency.

For this questionnaire examination, subjects were required to satisfy the following conditions: 1) The menstrual cycle was 25 to 38 days and duration of menses was 3 to 7 days for at least over the past year; Those who: 2) have not been diagnosed with dysmenorrhea or premenstrual syndrome; 3) have no history of gynecological diseases and do not regularly use drugs for menstrual symptoms; 4) do not use hormonal contraceptive pills or receive drug treatment for chronic diseases; 5) are not pregnant or in the lactation period; 6) have no history of mental disorders; 7) have been smoking at least ten cigarettes a day for at least the past year; 8) are in their twenties and have not married or given birth.

After obtaining consent from the subjects, a preliminary study (measurement for one menstrual cycle) was conducted. In the preliminary study, the same procedures as those in the main study were implemented to help the subjects learn the required techniques. As a result of the preliminary study, two women were diagnosed as having oligomenorrhea with a menstrual cycle of 39 days or longer, and one as probably having premenstrual syndrome. As a woman also withdrew from the study at this point, 29 subjects participated in the main study.

### Procedure

#### Definition of each phase of the menstrual cycle

Each phase of the menstrual cycle was determined during two menstrual cycles of the study period. The present study defined the period between the start and end of menstruation as the menstrual phase, the three-day period including the day in which an LH surge was identified plus the previous and following days as the ovulatory phase, the period between the menstrual and ovulatory phases as the follicular phase, and the period between the ovulatory and menstrual phases as the luteal phase.

#### Confirmation of ovulation

The subjects were asked to measure their basal body temperature (BBT) in every morning, and undergo a urinary LH test between the day after the end of menstruation and confirmation of an LH surge. The subjects recorded their results on a calendar every day.

#### Craving for smoking, depressiveness and menstrual phase-associated symptoms

Using the 100-mm Visual Analogue Scale (VAS), the subjects recorded their level of craving for smoking as a score from 0 [I do not feel like smoking] to 100 [I cannot help but smoke] every morning at the same time as BBT measurements.

A questionnaire survey was conducted to examine the level of depressiveness and menstrual phase-associated symptoms in the menstrual, follicular, and luteal phases. Each subject received the questionnaire form after consenting to the study, and sent it to us by mail after completing it. Dates for recording data in the form were determined based on the results of measurement of BBT and date of ovulation determined by a urinary LH surge. Symptoms in the menstrual phase were recorded within three days of the start of menstruation, and those in the follicular phase were recorded 3 to 5 days after the end of menstruation. Symptoms in the luteal phase were recorded between 10 days after confirming an LH surge and the start of the next menstruation.

The level of depressiveness was assessed using the Center for Epidemiologic Studies Depression (CES-D) Scale [14]. The CES-D is a four-grade (0 to 3 scores) scale for the self-assessment of depression, and includes 20 question items. The Japanese version of the CES-D was used in this study [15]. The total score is between 0 and 60. A subject with a total score of 16 or higher is determined as having depressiveness.

Symptoms associated with the menstrual cycle were assessed using the Menstrual Distress Questionnaire (MDQ) [16]. The MDQ is a four-grade (none - severe) scale and includes 47 symptoms and 8 sub-categories (the first: pain, second: impaired concentration, third: behavior change, fourth: autonomic reactions, fifth: water retention, sixth: negative affect, seventh: arousal, eighth: control). The reliability of the Japanese version of the MDQ used in the study has been established [17].

#### Tobacco consumption

To assess the amount of smoking, the subjects measured their breath CO concentration before bedtime every day and recorded it on a calendar along with the number of cigarettes smoked that day. Breath CO levels were measured using a MicroSmokerlyzer (Bedfont Scientific Ltd. UK) [detection range: 0 to 500 ppm; accuracy: ± 10%].

#### Other study items

The days on which the subjects drank alcohol were marked on a calendar during the study. The days the subjects had no chance to smoke were also marked in the same manner. Data from these days were excluded from the analysis.

#### Ethical considerations

The present study was conducted with the approval of the research ethics committees of Osaka University Graduate School of Medicine and Aino University. The anonymity of the subjects was preserved. The subjects are allowed to quit smoking if they so wished during the research period, and could receive treatment for nicotine addiction or symptoms associated with the menstrual phase. The subjects received book tokens worth 2,000 Japanese yen as remuneration.

#### Analysis methods

Using the descriptive statistics method, the attributes of research subjects, smoking status, and data on menstrual cycles were analyzed.

The number of cigarettes smoked and CO concentration during two menstrual cycles in the menstrual, follicular, and luteal phases were compared. The number of cigarettes smoked and CO concentration were analyzed using one-way repeated measures analysis of variance (ANOVA) and Tukey’s multiple comparison test.

The levels of craving for smoking, depression, and menstrual phase-associated symptoms during two menstrual cycles were compared among the menstrual, follicular, and luteal phases. Scores for each item were analyzed using one-way repeated measures ANOVA and Tukey’s multiple comparison test.

Pearson’s correlation coefficient was calculated between the amount of smoking (number of cigarettes smoked and CO concentration) versus levels of craving for smoking, depression, and menstrual phase-associated symptoms.

Statistical analysis software SPSS17.0 J for Windows was used for analyses. The significance level was set at p = 0.05 and p-values were based on 2-sided test.

## Results

Subjects were 15 full-time employees, 3 part-timers, and 11 students. Their age, smoking status and condition of menstruation are shown in Table 1.

**Table 1:** Characteristics of subjects

	**Mean (SD)**	**Range**
Age (years)	21.1 (1.7)	197–25
Daily number of cigarettes smoked	17.3 (5.2)	10.5–30.0
Years of smoking	3.6 (2.1)	1–10
FTND score	2.2 (2.1)	0–7
Duration of menstrual cycle (days)	30.5 (2.5)	37–34
Duration of follicular phase (days)	8.2 (2.1)	6–9
Duration of luteal phase (days)	12.6 (1.4)	12–15

*FTND*: Fagerström Test for Nicotine Dependency.

The number of cigarettes smoked and the CO concentration were significantly different among the menstrual phases (F(2,84) = 3.178, p = 0.007, partial eta-squared = 0.081; F(2,84) = 3.161, p = 0.019, partial eta-squared = 0.070, respectively) (Table 2). The number of cigarettes was greater (p = 0.028) and the CO concentration was higher (p = 0.034) in the luteal phase than in the follicular phase. A marked relationship was noted between the number of cigarettes smoked and CO concentration in the menstrual(r = 0.735,p < 0.001), follicular(r = 0.765,p < 0.001) and luteal phases(r = 0.810,p < 0.001).

**Table 2:** Amount of smoking (number of cigarettes smoked and CO concentration) in the menstrual, follicular and luteal phases

	**Menstrual mean (SD)**	**Follicular mean (SD)**	**Luteal mean (SD)**	**p-value**	**partial η**^**2**^
Number of cigarettes (per day)	16.1 (5.6)	14.7 (5.4)^1)^	18.3 (4.9)^1)^	0.007	0.081
Range	10.0–30.0	10.0–22.0	10.0–30.0		
CO concentration (ppm)	15.5 (5.8)	14.1 (6.8)^2)^	18.1 (5.1)^2)^	0.019	0.070
Range	3.7-28.0	4.0-30.0	11.0-36.0		

^1)^ p = 0.028, ^2)^ p = 0.034.

Data were analyzed by one-way repeated measures ANOVA followed by Tukey’s multiple comparison test.

**Figure 1:**
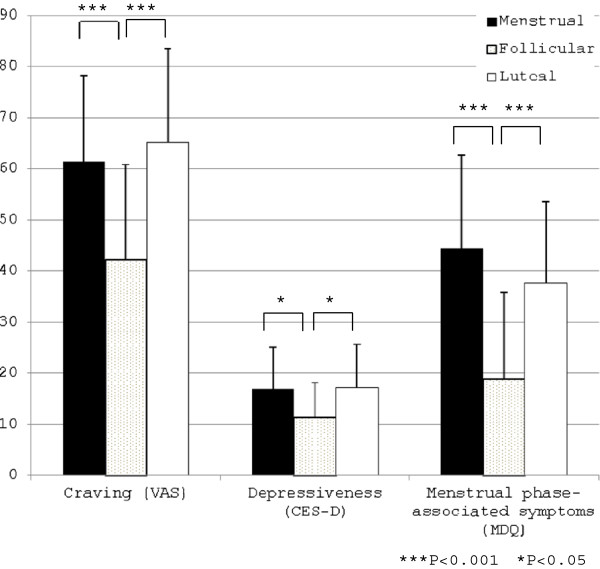
**Craving for smoking, depressiveness and menstrual phase-associated symptoms in the menstrual, follicular and luteal phases.** Scores of Craving (VAS) were 61.4 ± 16.7, 42.2 ± 18.6 and 65.0 ± 18.2 in the menstrual, follicular and luteal phases. Scores of Depressiveness (CES-D) were 16.9 ± 8.2, 11.3 ± 6.9 and 17.2 ± 8.4 in the menstrual, follicular and luteal phases. Scores of Menstrual phase-associated symptoms (MDQ) were 44.5 ± 18.2, 18.9 ± 17.0 and 37.7 ± 15.9 in the menstrual, follicular and luteal phases.

The levels of craving for smoking (VAS), depressiveness (CES-D) and menstrual phase-associated symptoms (MDQ) were significantly different among three phases (F(2,84) = 13.681, p < 0.001, partial eta-squared = 0.246; F(2,84) = 5.154, p < 0.001, partial eta-squared = 0.109; F(2,84) = 17.532, p < 0.001, partial eta-squared = 0.249, respectively). These symptoms in the menstrual and luteal phases were significantly higher than those in the follicular phase (Figure 1). In particular, the mean score for depressiveness was 16 points (the cut-off value in screening for depression) or higher in the menstrual and luteal phases.

The number of cigarettes smoked and CO concentration were correlated with the levels of craving for smoking (VAS), depressiveness (CES-D) and menstrual phase-associated symptoms (MDQ) in all phases except for MDQ scores in the follicular phase (Table 3). The correlation coefficients in the luteal phase were higher than those in the other two phases. Compared with the number of cigarettes smoked, the CO concentration showed a higher correlation coefficiency to the CES-D and MDQ scores in all phases, whereas it showed lower correlation coefficiency to the levels of craving for smoking in the menstrual and follicular phases.

**Table 3:** Correlation coefficiency between thpfe amount of smoking and levels of craving for smoking, depression, and menstrual phase-associated symptoms in the menstrual, follicular and luteal phases

		**Menstrual**		**Follicular**		**Luteal**	
		**r**	**95% CI**	**r**	**95% CI**	**r**	**95% CI**
Craving (VAS)	Number of cigarettes	0.611^***^	0.20–0.84	0.598^***^	0.18–0.83	0.718^***^	0.38–0.89
	CO concentration	0.530^**^	0.09–0.80	0.430^*^	0.08–0.69	0.811^***^	0.55–0.93
Depressiveness (CES-D)	Number of cigarettes	0.387^*^	0.02–0.66	0.374^*^	0.01–0.65	0.546^**^	0.24–0.77
	CO concentration	0.490^**^	0.03–0.78	0.484^**^	0.02–0.78	0.607^***^	0.20–0.84
Menstrual phase-associated symptoms(MDQ)	Number of cigarettes	0.405^*^	0.05–0.67	0.097	−0.28–0.45	0.530^**^	0.08–0.80
	CO concentration	0.488^**^	0.03–0.78	0.274	−0.10–0.58	0.588^**^	0.17–0.83

^*^ p < 0.05, ^**^ p < 0.01,^***^ p < 0.001.

95% CI: 95% confidence interval.

## Discussion

To accurately examine the relationships between the amount of smoking versus levels of craving for smoking, depression, and menstrual phase-associated symptoms among the menstrual, follicular, and luteal phases, we improved the methodological issues described in former studies. The first problematic issue is the definition of the menstrual phases. We clearly defined the menstrual phases for each subject, as described in Materials and Methods. Benowitz et al. reported that estrogens accelerated the nicotine metabolism by comparison between women versus men [18], but the activity of cytochrome P450 2A6, which is primarily responsible for nicotine metabolism, was not affected by menstrual cycle phase [19]. Since the level of sex hormones fluctuated during the menstrual cycle, the association of ovarian hormones and smoking behavior has been examined in recent psychological studies [20-22]. These studies attempted to directly examine the pharmacological effect of progesterone and/or estrogen (e.g., hormonal contraception) on women’s sensitivity to nicotine, but such effects on smoking behavior are also unclear. As oral contraceptives were reported to accelerate the nicotine metabolism [18], women using hormonal contraceptives were excluded. Secondly, there is an issue with the method for measuring breath CO and urinary cotinine levels as objective indices of the amount of smoking. In previous studies [6,7,9,10], these biomarkers were measured once in each menstrual cycle when the subjects visited a hospital or research institution. Since the amount of smoking can vary every day, measurement should be performed much more frequently. In the present study, breath CO levels were measured every day to reduce such bias. Additionally, Craig et al. [2] mentioned that women smokers tended to drink alcohol before a menstrual period, which could have caused an increase in the amount of smoking in the luteal phase. In this study, the data on days when the subjects drank alcohol or had no chance to smoke were excluded from the statistical analysis. We then concluded that the number of cigarettes smoked by young Japanese women and their breath CO levels significantly increased in the luteal phase compared with the follicular phase.

In this study, the level of craving for smoking in young Japanese women smokers in the menstrual and luteal phases was higher compared to the follicular phase without smoking cessation. Just as a long period of smoking cessation induces withdrawal symptomatology, a strong craving for smoking is sometimes noted even in smokers. Previous studies reported that the level of craving for smoking was higher and the power of concentration was decreased more in the menstrual phase compared with the follicular phase [6], and that withdrawal symptoms worsened in the luteal phase [6,7,23]. The present study also identified a strong positive correlation between the levels of craving for smoking and the amount of smoking in the luteal phase.

The levels of depressiveness in young Japanese women smokers in the menstrual and luteal phases were also higher compared to the follicular phase. The subjects of the study did not have premenstrual syndrome (including premenstrual dysphoric disorder) or dysmenorrhea. Nevertheless, the mean CES-D scores in the menstrual and luteal phases were 16 or higher - an indication of depression. This suggests that the level of depressiveness in young Japanese women smokers changes according to the menstrual cycle, and that a large number of these smokers develop depressiveness in the luteal phase. When screening the level of depressiveness in women smokers, careful attention should be paid to the relationship between the time of measurement and the menstrual cycle. There was a stronger correlation between the amount of smoking and the level of depressiveness in the luteal phase compared with other phases. The relationship between smoking and depression is well-recognized [24,25] and further studies should be conducted to determine whether depression in the luteal phase causes an increase in the amount of smoking or smoking increases the level of depression.

MDQ scores were high in both the menstrual (44.5 ± 18.2) and luteal (37.7 ± 15.9) phases. Our previous study reported that young Japanese female smokers showed levels that could be considered symptoms (MDQ score; 34.9 ± 19.2 in the menstrual phase, 39.5 ± 24.9 in the luteal phase) [12]. In the menstrual and luteal phases, a moderate positive correlation was noted between MDQ scores and the amount of smoking. However, there was no significant correlation between them in the follicular phase. Although the MDQ subscale includes “negative emotions” similar to “depressiveness”, the correlations with the amount of smoking differed from each other in the follicular phase. This suggests that factors other than depressiveness may also contribute to the correlation with the amount of smoking in the menstrual and luteal periods.

### Limitations

In this study, the subjects were 29 young Japanese female smokers aged from 19 to 25 years old. Smoking behavior and the menstrual phase-associated symptoms vary among countries, cultures and ages. The correlations between the amount of smoking and several symptoms shown in the study were possibly limited to these study subjects. Generalization of our results should be made only with caution. In addition, no cause-effect relationship between the amount of smoking and these symptoms has been determined. Further studies will be needed in the future.

## Conclusions

In the menstrual and luteal phases, young Japanese women smokers increased their amount of smoking and suffered from greater craving for smoking, depressiveness and menstrual phase-associated symptoms compared with the follicular phase. The amount of smoking was correlated with these symptoms, but their cause-effect relationship has not been determined yet.

## Competing interests

The authors declare that they have no competing interest.

## Authors’ contribution

HS and KO designed the study. HS collected the data. HS and KO conducted the data analysis. HS wrote the first draft of the manuscript. KO completed the manuscript for submission. Both authors read and approved the final manuscript.

## Pre-publication history

The pre-publication history for this paper can be accessed here:

http://www.biomedcentral.com/1472-6874/13/10/prepub
